# Antibacterial Silver

**DOI:** 10.1155/MBD.1994.467

**Published:** 1994

**Authors:** Julia L. Clement, Penelope S. Jarrett

**Affiliations:** 1 School of Chemistry and Applied Chemistry University of Wales College of Cardiff P.O. Box 912 Cardiff CF1 3TB United Kingdom

## Abstract

The antibacterial activity of silver has long been known and has found a variety of applications because its toxicity to human cells is considerably lower than to bacteria.   The most widely documented uses are prophylactic treatment of burns and water disinfection. However, the mechanisms by which silver kills cells are not known. Information on resistance mechanisms is apparently contradictory and even the chemistry of Ag^+^  in such systems is poorly understood.

Silver binds to many cellular components, with membrane components probably being more important than nucleic acids. It is difficult to know whether strong binding reflects toxicity or detoxification: some sensitive bacterial strains have been reported as accumulating more silver than the corresponding resistant strain, in others the reverse apparently occurs. In several cases resistance has been shown to be plasmid mediated. The plasmids are reported as difficult to transfer, and can also be difficult to maintain, as we too have found. Attempts to find biochemical differences between resistant and sensitive strains have met with limited success:  differences are subtle, such as increased cell surface hydrophobicity in a resistant *Escherichia coli.*

Some of the problems are due to defining conditions in which resistance can be observed. Silver(I) has been shown to bind to components of cell culture media, and the presence of chloride is necessary to demonstrate resistance. The form of silver used must also be considered. This is usually water soluble AgNO_3_, which readily precipitates as AgCl. The clinically preferred compound is the highly insoluble silver sulfadiazine, which does not cause hypochloraemia in burns. It has been suggested that resistant bacteria are those unable to bind Ag^+^ more tightly than does chloride. It may be that certain forms of insoluble silver are taken up by cells, as has been found for nickel. Under our experimental conditions, silver complexed by certain ligands is more cytotoxic than AgNO_3_, yet with related ligands is considerably less toxic. There is evidently a subtle interplay of solubility and stability which should reward further investigation.


### ANTIBACTERIAL SILVER

Julia L. Clement and Penelope S. Jarrett

School of Chemistry and Applied Chemistry, University of Wales College of Cardiff, P.O. Box 912, Cardiff CF1 3TB, U.K.

The antibacterial activity of silver has long been known and has found a variety of applications because its toxicity to human cells is considerably lower than to bacteria. The most widely documented uses are prophylactic treatment of burns and water disinfection. However, the mechanisms by which silver kills cells are not known. Information on resistance mechanisms is apparently contradictory and even the chemistry of $Ag^+$ in such systems is poorly understood.

Silver binds to many cellular components, with membrane components probably being more important than nucleic acids. It is difficult to know whether strong binding reflects toxicity or detoxification: some sensitive bacterial strains have been reported as accumulating more silver than the corresponding resistant strain, in others the reverse apparently occurs. In several cases resistance has been shown to be plasmid mediated. The plasmids are reported as difficult to transfer, and can also be difficult to maintain, as we too have found. Attempts to find biochemical differences between resistant and sensitive strains have met with limited success: differences are subtle, such as increased cell surface hydrophobicity in a resistant *Escherichia coli.*

Some of the problems are due to defining conditions in which resistance can be observed. Silver(I) has been shown to bind to components of cell culture media, and the presence of chloride is necessary to demonstrate resistance. The form of silver used must also be considered. This is usually water soluble $AgNO_3$, which readily precipitates as AgCl. The clinically preferred compound is the highly insoluble silver sulfadiazine, which does not cause hypochloraemia in burns. It has been suggested that resistant bacteria are those unable to bind $Ag^+$ more tightly than does chloride. It may be that certain forms of insoluble silver are taken up by cells, as has been found for nickel. Under our experimental conditions, silver complexed by certain ligands is more cytotoxic than $AgNO_3$, yet with related ligands is considerably less toxic. There is evidently a subtle interplay of solubility and stability which should reward further investigation.

### Introduction

The antimicrobial activity of silver appears to have been known since early in recorded history. Herodotus describes how the King of Persia, when going to war, among his provisions took boiled water stored in flagons of silver (1,2). The first modern description of this effect was given by Raulin in 1869, who observed that *Aspergillus niger* could not grow in silver vessels (3,4). He was somewhat upstaged by the Swiss botanist von Nägeli (3,5) who devised the term "oligodynamic" to describe any metal which exhibits bacteriocidal properties at minute concentrations ("oligos", small + "dynamis", power). He was studying silver, and it is especially true of this metal, although copper and tin also have oligodynamic activity. Von Nägeli distinguished between "oligodynamic" death, and "ordinary" poisoning at measurable concentrations. This terminology seems to have contributed to much confused thinking about the means and mechanism(s) by which silver kills bacteria, most of which have been well documented by Romans (6), but the word "oligodynamic" still appears in quite modern textbooks (7).

There are several more recent discussions of the biological activity and importance of silver from different points of view (8-12).

### Water Sterilization

The idea of oligodynamic activity has been behind the development of many antimicrobial processes and products. One of the earliest and most studied was "Katadyn silver" (13), described as a spongy preparation of metallic silver, containing a small amount of added palladium or gold. The intention was to maximise the silver surface area and thus the area in contact with the water. It has been used to coat sand, the insides of flasks, or other materials, and to impregnate filters (14). The wide range of reported activity for Katadyn silver (0.006 ppm-0.5 ppm) illustrates a major problem for those working with silver: the concentrations required for antibacterial effect are generally low and are very dependent upon the conditions used. Some workers recognised the importance of the medium. The activity of Katadyn rings is inhibited by the presence of organic matter and inorganic substances (15), that of Katadyn flasks is inhibited by milk (16) and even Dresden tap water was less bacteriocidal than distilled water containing the same concentration of silver(I) (17). The same workers (17) also demonstrated the inhibitory effects of colloids, low temperature, and storage in glass containers.

Despite these problems and its relatively high cost, silver is still an attractive option for water sterilization since it is active at low concentrations for long periods, is odourless and of extremely low toxicity to humans. Various methods have been used to introduce silver ions into the water, including direct addition of soluble silver(I) salts, inorganic composites and electrical methods such as "Electro-Katadyn" (6). Popular applications are to drinking water (19) and swimming pools, although Mallman (20) concluded that Electro-Katadyn was not an appropriate treatment since the total bacterial counts were too high, despite finding that the numbers of *E. coli* were reduced sufficiently and the remaining bacteria were not pathogenic. Silver is still promoted as a swimming pool disinfectant. A recent suggestion is to combine electrolytically generated copper(I) and silver(I) with a chlorine treatment, thereby reducing the concentration of irritating chlorine which needs to be used (18).

### Therapeutic Applications

Silver nitrate was introduced by Credé in 1884 for the prevention of ophthalmia neonatorum, and until recently most states of the USA required that a few drops of a 1% $AgNO_3$ solution be instilled in infants' eyes immediately after birth for the same purpose (6). Simple silver(I) salts give rise to high concentrations of $Ag^+$ in solution, which is precipitated by chloride and proteins, giving rise to astringent effects. This principle is applied in caustic silver nitrate pencils for the cauterization of small wounds and removal of granulation tissue (21). Such effects, however, are usually undesirable for an antibacterial agent and a variety of colloidal preparations, including silver proteinate, were developed, mainly for topical use (6,21). These have been supplanted by other antibiotics.

Although $Ag^+$ is locally astringent, silver is not poisonous to mammals. Romans (6) has summarised much of the data. Excessive amounts of silver compounds or long term treatment with silver(I) (22) can cause argyria. This is discolouration of the skin or tissues which is generally irreversible but does not seem to be harmful. Indeed, a study of many cases could find no cellular reactions to the deposited silver (23). $AgNO_3$ does inhibit respiration of guinea-pig ear skin in tissue culture, but at about 25 times the minimal concentration which killed *Pseudomonas aeruginosa* (60). Canadian women were found to ingest 7.1 mg daily from their food, with no apparent ill-effect (24). Acute oral toxicity ranges from 2 to 30g (8).

At present, the major therapeutic use of silver(I) is in the topical chemoprophylaxis of burns. Silver nitrate had been used at high concentrations, but was reintroduced at lower concentration in 1965 (25). A controlled trial using compresses containing 0.5% silver nitrate on severely burned patients showed its effectiveness at preventing infection by *Pseudomonas aeruginosa* (76) and the related septicaemia (26). The use of $AgNO_3$ was associated with reduced mortality (27). In common with other topical antibiotics of the time, there were problems. The particular problem of silver nitrate was that the hypotonic solution caused electrolyte alterations which had to be treated with supplementary calcium, sodium and potassium (28). This led Fox (29) to develop silver sulfadiazine, a water insoluble salt which dissolves only slowly in biological fluids. The intention was that low concentrations of $Ag^+$ would be attained, chloride would not be readily precipitated and antibacterial activity would be maintained. Controlled trials confirmed its usefulness (77) and it is now the treatment of choice.

Sulfadiazine is a sulfonamide and is itself an antibiotic. However, the sulfonamide antagonist para-aminobenzoic acid did not inactivate silver sulfadiazine (AgSu) (30). Further evidence that the silver was the active component came from the observation that sulfadiazine alone was ineffective at concentrations at which AgSu inhibited bacterial growth (31,43). Indeed, sulfadiazine exhibited specific synergism in combination with subinhibitory levels of AgSu (31), which suggests that the two agents kill bacteria by different mechanisms. Furthermore, Ag resistant bacteria from AgSu treated burns are not necessarily Su resistant, nor vice versa (31,81). Binding experiments provided circumstantial evidence for the importance of silver. At inhibitory concentrations of $Ag^{35}Su$ or

$^{110m}$AgSu, up to 20% of the radioactive silver was bound by cultured cells, whereas the uptake of radiolabelled sulfadiazine was negligible (32). Incubations of AgSu with NaCl, nutrient broth, DNA, human serum and bacteria provided experimental support for the hypothesis that AgSu provided slow sustained delivery of Ag (31). Some salts, such as $AgNO_3$, dissolved immediately and the silver would be 'lost' as AgCl or other precipitates. Others showed negligible dissociation over 40 hours, releasing very little silver, while AgSu dissociated at an intermediate rate and maintained a roughly constant concentration of silver (31).

Complexation of silver by biological molecules

The stability of silver(I) complexes varies with the ligand donor atom: N<<P>As>Sb; O<<S~Se~Te; F<Cl<Br<I and Ag(I) has been categorized as a class b or 'soft' acid (33). Although it binds most strongly to P and S donor ligands, it has an extensive chemistry with N (in particular) and O donors(33), and appears to bind to almost every biological molecule tested. Silver(I) has long been known to form complexes with amino acids (33). It is used to stain proteins in polyacrylamide gels, being particularly useful when small amounts of protein need to be detected since it is more sensitive than techniques based on Coomassie Blue. When various polyamino acids were tested, polyhistidine stained the most deeply (34). In certain proteins, silver has been shown to bind at specific sites: forming $Ag_6$, $Ag_{12}$ and $Ag_{18}$ clusters with a rabbit metallothionein (35) and displacing Cu(I) from its binding site in reduced azurin from *Ps. aeruginosa* (36).

Given the propensity of silver(I) to complex with so many molecules, it is obviously important to consider what interactions may occur between the medium and added $AgNO_3$. Decreased bacteriocidal activity in certain media was observed by those working on water sterilization (10,11,12) (see above), and the effects of different nutrient media have been specifically investigated (37). Three common agar media differed in their capacity to reduce the bacteriostatic effects of $AgNO_3$. Since broth and agar forms did not differ, the effect was attributed to soluble compounds of the media rather than the agar (37). Two silver(I) phosphine complexes exhibited antifungal activity in a "defined" medium, which was lost in a "complex" medium (38), and the toxicity of one of these complexes toward tumour cells *in vitro* was reduced by a factor of five by the presence of foetal calf serum in the medium. These effects were attributed to reaction with serum proteins and other components of the media (38). The component which has given rise to the most comment is chloride, frequently present at up to 171mM ($10gl^{-1}$ of NaCl) in nutrient broths. The effects of chloride will be discussed in more detail below.

Silver(I) also forms complexes with some nucleosides and nucleotides at neutral pH (probably binding at N-7 of the purine base) (39), with RNA (40), and with DNA (31,41,42). Most such experiments utilise $AgNO_3$. Rosenkranz and Rosenkranz (42) compared the interactions of $AgNO_3$ and AgSu with DNA and concluded that they were different. One of the effects of Ag(I) binding to DNA is to increase the sedimentation coefficient (41,42). Wysor and Zollinhofer (43) reported that the sedimentation coefficient of DNA from *Ps. aeruginosa* treated with AgSu was increased relative to DNA from untreated bacteria, while Rosenkranz and Carr (44) found no such difference in the DNA from their treated and untreated *Ps. aeruginosa.* They further reported that bacteria deficient in DNA polymerase were not more sensitive to AgSu than was the parent strain (44). Fox, however, continued to emphasise the importance of DNA binding. In a series of experiments using radiolabelled silver and sulfadiazine ($^{35}S$) he showed that only the Ag from AgSu became associated with isolated DNA (31). Using very low concentrations of AgSu, sufficient only to delay rather than prevent growth, he found that Ag binding to RNA and cell residue (proteins and polysaccharides) rose to a maximum, then stayed stable up to 4 hours. In contrast, binding to DNA reached a maximum and then decreased again (32). This decrease seemed to coincide with the resumption of bacterial growth. Since this work there has been very little interest in silver interactions with nucleic acids.

Localization of silver in cells.

In his experiments treating *Ps. aeruginosa* with sublethal concentrations of AgSu, Fox found that less than 0.5% of the silver was in the lipid fraction, up to 3% in the RNA fraction, up to 12% in the DNA fraction and the remainder was bound to the cell "residue" (proteins and polysaccharides) (32). Similar observations have been made by many other workers. Rosenkranz and Carr found that most silver was associated with the cell membrane (and proteins), and some with the cell wall (43). They further examined AgSu treated bacteria by electron microscopy, and observed them to be distorted in shape, with surface blebs (45). A strain resistant to AgSu did not display surface blebs. The same was true of sensitive and resistant *Enterobacter cloacae* (66). $AgNO_3$ caused aggregation of nuclear material, but no blebbing (45). Longer exposures, however, did cause blebbing (46).

Binding of silver by bacteria is also of interest to those hoping to recover metals from industrial effluents and waste materials (47,48,49) or by leaching from minerals (50) (Table I). Such workers have also looked at the localization of silver, but usually treat the bacteria with much more concentrated solutions than the AgSu work referred to above (mM rather than μM). The distribution of silver in *Citrobacter intermedius* was 5% in the cytoplasm, about 27% in the cell membrane and about 68% in the cell wall fraction (49); electron micrographs showed large electron dense granules associated with the cell envelope (49). Thiobacilli collected from sulphide leaching systems were also covered with electron-dense granules (50). Microprobe analysis showed they contained silver and sulphur, and electron diffraction suggested the mineral acanthite ($Ag_2S$) (50). This result may have been very particular to this system, since the electron-dense particles of a silver resistant pseudomonad (treated with *ca.* 0.6 mM $AgNO_3$) were not $Ag_2S$ nor AgCl as demonstrated by energy-dispersive X-ray detection (51). The author suggested Ag(0), Ag(I) or silver oxide. Blebbing was not reported at these concentrations.

Although most silver is undoubtedly associated with the cell wall, the membrane and proteinaceous cellular appendages (pilae, flagellae), it is difficult to establish the relative importance of this binding. $^{110}Ag$ from $AgNO_3$ could not be washed off *Ps. aeruginosa* using nutrient broth, but 0.1% $AgNO_3$ completely removed it (60). Hughes and coworkers (53) have described two types of binding sites: a low capacity, high affinity binding site (probably intracellular) from which $Ag^+$ is not released by acid washing, and higher capacity, lower affinity sites (probably on the surface) from which $Ag^+$ is released by acid washing. They suggest $Ag^+$ is taken up in a metabolism-independent process and bound at specific intracellular sites. Saturation of these allows surface binding of $Ag^+$.

The consensus seems to be that surface binding and damage to membrane function are important in the killing of bacteria by silver. This is to some extent supported by reports of the metabolic effects of silver(I). It inhibits several oxidative enzymes (61), yeast alcohol dehydragenase (62), the uptake of succinate by membrane visicles (63) and the respiratory chain of *E. coli*, although this last has since been suggested to be due to the nitrate counter ion (65). It is also reported to cause metabolite efflux (83). The early workers on AgSu were particularly interested in macromolecular synthesis: it was reported both that protein, RNA and DNA syntheses are blocked in AgSu treated bacteria (44) and that protein and nucleic acid syntheses are not inhibited in cell free systems (43).

### Bacterial Resistance to Silver

The widespread use of AgSu led inevitably to the isolation of silver resistant bacteria from burn units (Table II. Some reports of silver resistance are not included in the table (75-80)). This was unwelcome to both physicians and their patients; three of the first cases to be described died after infection by a multiply resistant *Salmonella typhimurium* (67). There have been some encouraging observations, however. Resistance is often reported to be unstable or difficult to maintain (70,74,79) and is also difficult to transfer (67,78). Although it could be transferred (at very low frequency: $10^{-6}$-$10^{-5}$) from the S. *typhimurium* (67) mentioned above, a further 13 strains from the same laboratory were not able to donate resistance (69). Resistance was only transferred from *Ps. stutzeri* AG259 when mobilized by a conjugative plasmid (52). It was not transferred from *E. coli* R1 (55) nor three other resistant *E. coli* (57) by membrane filter mating, nor from *E. coli* R1 by artificial competence, high-voltage electroporation nor plasmid mobilization (57). It was eventually transferred to *E. coli* C600 using a Tn5-Mob transposon (59). High-voltage electroporation succeeded in transferring resistance from *Ps. stutzeri* AG259 to *Ps. putida* CYM318 (58). Membrane filter mating was sufficient to transfer resistance from *Acinetobacter baumanii* to an *E. coli* K12, and thence to another *E. coli* K12, but at frequencies of only about 1 x $10^{-6}$ in each case (74). Levels of resistance in recipient bacteria have been reported as lower than those for the donor (67).

The frequency of occurrence of silver resistance seems variable: 2 (probably the same strain) out of 72 *Enterobacter* species (66); 3 isolates (same strain) out of 19 *salmonellae*; zero out of 154 *E. coli* (67); 5 out of 168 *Klebsiellae* and 6 out of 119 *Enterobacter* species (67); 14 out of 1000 isolates

Table I Accumulation of silver by bacteria

| Organism | Silver accumulated (mg) by dry weight of bacteria (g) | Reference |
|---|---|---|
| *Ps. maltophilia, Staphylococcus aureus* and a coryneform organism | >300 | 47 |
| *Thiobacillus ferrioxidans and T. thiooxidans* | 250 | 50 |
| Pseudomonad | 300-390 | 51 |
| *Ps. stutzeri* AG259 | 2 | 52 |
| Unidentified | 23 | 48 |
| *Escherichia coli* | 67 | 53 |
| *Citrobacter intermedius* | 28 (growing)<br>44(non-growing) | 49 |
| *Ps. stutzeri* AG259 | 0.8 | 54 |
| *Escherichia coli* R1 and S1 | 390-2,500 | 55 |
| Coryneform organism | 2,000 | 56 |
| *Escherichia coli* R1 | 2 | 57 |
| *Escherichia coli* S1 | 12 | |
| *Ps. stutzeri* AG259 | 1,170 | 58 |
| *Ps. putida* CYM318(±pKK1) | 2,000 | |
| *E. coli* R1 and C600(pJT1, PJT2) | 2,700-3,100 | 59 |
| *E. coli* C600 | 7,300 | |

[a]mg/g cell protein

Table II Bacteria reported to be resistant to silver[a]

| Organism (number of strains) | MIC[b] resistant strain(s) | MIC[b] sensitive strain(s) | References |
|---|---|---|---|
| *Enterobacter cloacae*, *vs.* AgSu[c] | >1.1 mM | 9 - 14 mM | 66 |
| *Salmonella typhimurium*[d] | 10 mM | 0.6 mM | 67 |
| *Enterobacter* sp. (7) | 5 - 20 mM | - | 69 |
| *Klebsiella* sp. (5) | 5 - 20 mM | - | |
| *Pseudomonas aeruginosa* | 5 mM | - | |
| *Escherichia coli* (13) | 0.25 - >5 mM | - | 70 |
| *Enterobacter cloacae* (4) | 0.5 - 5 mM | - | |
| *Klebsiella pneumoniae* (8) | 0.5 - 5 mM | - | |
| *Proteus mirabilis* | 0.25 mM | - | |
| *E.coli*[d] J62 (±pSC35), *vs.* AgSu | ~6 µM | ~0.2 µM | 71 |
| *E.coli* J62 (±pSC35), *vs.* $AgNO_3$, no $Cl^-$ | ~4 µM | ~0.4 µM | |
| *E.coli* J62 (±pSC35), *vs.* $AgNO_3$, with $Cl^-$ | > 50 µM | ~0.4 µM | |
| *Pseudomonas* (4) | >0.6 mM | ~0.06 mM | 51 |
| *Ps. stutzeri*[d] AG259 and AG256, with $Cl^-$ | >25 mM | 0.25 mM | 52 |
| *Ps. stutzeri* AG259 and AG256, no $Cl^-$ | 0.8 mM | 0.25 mM | |
| *K. pneumonia*, with or without $Cl^-$ | >0.5 mM | 0.1 mM | 72 |
| *Ps. putida*[d] CYM318(±pKK1) | >0.5 mM | >0.05 mM | 58 |
| *E.coli*[d] R1 and S1 (±pJT1) | >1 mM | 0.5 mM | 55 |
| *E.coli*[d] C600 (±pJT1, pJT2) | >0.5 mM | ~0.1 mM | 59 |
| *Ps. stutzeri*[d] AG259 and JM303 | >0.5 mM | 0.05 mM | 73 |
| *Acinetobacter baumanii*[d] BL88 | 1 mM | | 74 |

a Silver as $AgNO_3$, unless otherwise stated b MIC = minimum inhibitory concentration c AgSu = silver sulfadiazine d Identified as containing plasmids.

(69). These were all from hospitals, mainly burns units. Frequencies in city and hospital sewage were 0.008-0.028 (percent of total viable count) (69), but were very much higher in film reprocessing sludge: 94% (69). The other, less commonly studied, sources of silver resistant bacteria are silver mines (48,52).

The isolation of resistant strains has been enormously useful to experimentalists, as it has allowed good comparative experiments to be done. The enormous range of figures quoted in Tables I and II undoubtedly reflects differences in experimental procedure and conditions, as well as differences between bacterial species. Nevertheless it is difficult to understand how such different results can be given for the same species by the same workers (54,58,55,57) without even commenting on them. Effects of the medium used have already been mentioned (10,11,12,37,38). A concentration quoted as the MIC of a sensitive strain in one experiment might kill a resistant strain in another experiment (Table II), and it is obviously important to compare sensitive and resistant strains of the same organism under the same conditions in order to uncover real differences.

Possible Resistance Mechanisms

Accumulation of metal by bacteria has often been implicitly assumed to be related to resistance to, or at least "tolerance" of, the metal. Comparisons of silver sensitive and resistant bacteria show that this is not necessarily so. Silver resistant *Klebsiella pneumoniae* took up 3-4 times less silver than the sensitive strain (72); silver resistant *E. coli* R1 accumulated 5 times less silver than *E. coli* S1 (57) (this could also be seen by energy dispersive X-ray analysis but not by measurements with an $Ag^+$-specific ion electrode (55)). Transfer of plasmids pJT1 and pJT2 to *E. coli* C600 conferred resistance, and the recipient displayed decreased accumulation of Ag (59). Accumulation behaviour, however, may be species specific. The pattern of silver uptake by resistant *Acinetobacter baumanii* (pUP188) differed from that of a transformed *E. coli* (pUP188), the latter appearing to take up then efflux silver (74). *Ps. putida* CYM318, with or without pKK1 from *Ps. stutzeri* A259, demonstrated very similar levels of silver accumulation (58). And *Ps. stutzeri* behaves differently from all other species studied: electron microscopy and energy-dispersive X-ray analysis showed dense silver deposits associated with the cell surfaces of silver resistant AG259 but not silver sensitive JM303 (82). There are some suggestions that bacterial accumulation of silver may be an energy-dependent process (82,54). This variable pattern of behaviour emphasises the importance of comparing sensitive and resistant organisms only when they are of the same species.

Many possible mechanisms for bacterial resistance to silver have been proposed, but much work, mainly by Trevors and co-workers, has failed to produce strong support for any one of them. A resistant *Pseudomonas* produced a volatile compound which reduced Ag(I) (51), but when resistant and sensitive strains of *Ps. stutzeri* were compared, their reductive capacities were indistinguishable (52). Both *E. coli* R1 and *E. coli* S1 produced $H_2S$ (detected by blackening of lead acetate paper) (55). When $H_2S$ and intracellular acid labile SH were assayed for, in the absence of $AgNO_3$, the silver resistant *E. coli* R1 produced >30% more of each than did *E. coli* S1 (57). The positive control, *E. Coli* pIP, produced more $H_2S$ than *E. coli* R1, but was sensitive to silver (57). There are many difficulties and possible interferences in determinations of thiol concentrations (73), but it seemed that silver resistant *Ps. stutzeri* AG259 produced less $H_2S$ but had higher intracellular acid-labile sulphide levels than silver sensitive *Ps. stutzeri* JM303 (73). Blackening of colonies is often reported (51,52,55,74).

The possibility that resistant strains produced a silver binding protein, which would sequestor the metal, was investigated. In the range 14-200 kDa no new proteins were detected by SDS-PAGE from *E. coli* R1 (55). Indeed, it seemed to lack two outer membrane proteins found in *E. coli* S1 (55). HPLC protein analysis of cell-free extracts showed some low molecular weight fractions were reduced in concentration when either *E. coli* R1 or S1 were grown in the presence of sufficient silver(I), suggesting it inhibited protein synthesis (57). In addition, in the absence of silver(I), *E. coli* R1 lacked several fractions present in *E. coli* S1, and displayed higher cell surface hydrophobicity (57). *Ps. stutzeri* strains produced similar proteins in the absence of silver, but the silver-sensitive JM303 produced high molecular weight proteins during exposure to silver (73). No 6-8 kDa proteins (typical of metallothionein) were detected, and none of the proteins contained silver (73). There was no discernible difference between $^{31}P$-NMR spectra of these strains, and intracellular polyphosphate was not thought to be involved in binding silver (73). Some authors have suggested that silver may be toxic by more than one mechanism (83), and that there may be more than one system of resistance (72).

A particularly interesting aspect of the influence of the testing medium has been highlighted by Simon Silver. The presence of chloride reduces the toxicity of silver only to resistant strains, not to sensitive ones (71) (Table II). Although NaCl was reported not to affect the sensitivity of *K. pneumoniae* (72), the effect has been confirmed by other workers (52,54,66) and in our own work (see below). It has been hypothesized that resistant strains cannot compete with AgCl for the silver (71). This would seem to agree with the observation that most resistant strains accumulate less silver than their sensitive counterparts, and perhaps with resistant strains lacking outer membrane proteins, as found by Trevors and co-workers (57,73). It may also help in understanding the variability in older work with mixtures of bacteria: only the resistant components would be affected by the concentration of chloride. AgSu toxicity seems less affected by chloride than $AgNO_3$ toxicity (71), but both can be affected by the presence of bovine serum albumin, with resistant and sensitive strains of *E. coli* again responding differently (84).

A further complicating factor is the concentration of copper, likely to be an impurity in growth media at ~0.3mM (53). Growth of *E. coli* K12 was found to be dependent on the Ag/Cu ratio, rather than concentration of $Ag^+$ alone (53) and accumulation of silver was also affected by the presence of copper (53,54). That Ag(I) can displace Cu(I) from its binding site in azurin has already been mentioned (36).

Physical and Chemical Properties of Silver Complexes

The structure of crystalline AgSu has been determined from X-ray diffraction experiments (85,86). Each silver is coordinated to three sulfadiazine molecules, and each sulfadiazine to three silvers, to form polymeric chains extending through the crystal. As expected for a polymer, it is very insoluble: the solubility in water at pH 7 is about 0.1 mg/100ml (2.8mM) (87). In addition to pH, the solubility also depends on ionic strength and the presence of other complexing agents such as $NH_3$ (87). Polymeric structures and low solubility are extremely common among silver complexes (33). This makes it difficult to evaluate antibacterial activity experimentally. It is also the reason why therapeutic use of silver(I) is confined to topical application, and there are no systemic silver(I) antibiotics.

Our approach has been, for the first time, to compare the toxicity of various silver(I) compounds to silver resistant and silver sensitive strains of the same species, which were otherwise biochemically identical. This has only become possible in recent years as plasmid bearing strains have been isolated and characterised. The silver(I) compounds too are well characterised, with known structures and properties. This permits us to monitor their reactivity and solubility, and to try and relate their chemical properties to their antibacterial effects. In so doing, we may hope to understand more about resistance mechanisms, and also discover which types of compound may allow us to circumvent such systems.

### Materials and Methods

Synthesisl

Silver nitrate, 1,2-bis-(diphenylphosphino)ethane (dppe), 1,2-bis(diphenylphosphino)ethene (dppey), triphenylphosphine ($PPh_3$) and 2,2'-bipyridine (bipy) were obtained from Aldrich Chemical Co; 1,2-bis(diethylphosphino)ethane (depe) from Strem. The complexes were synthesized by literature methods (88,89,90) and gave satisfactory elemental analyses and (in the case of the phosphine complexes) $^{31}P$-{$^1H$} NMR spectra.

NMR Measurements

$^{31}P$-{$^1H$ NMR } spectra were recorded on a Jeol FX90Q Spectrometer, operating at 36.15 MHz, in 5-mm tubes and referenced to an external sample of 85% $H_3PO_4$. The phosphine complexes were dissolved in N,N'-dimethylacetamide (DMA), with $D_2O$ in a capillary insert as lock. $^{31}P$-{$^1H$} NMR

spectra were recorded and then additions made of either NaCl (10 mol. equiv.) dissolved in water or dithiothreitol (DTT) in DMA or sodium β-D-thioglucose dihydrate (NaSGlu, 1 and 3 mol. equiv.) dissolved in methanol; apart from $[Ag(dppey)_2]NO_3$, to which 1 and 1.5 mol. equiv. of thiols were added. $^{31}P$-{$^1H$} NMR spectra of the solutions were recorded again after the additions, with any solid precipitates having been removed.

### Antibacterial Activity

*Escherichia coli*, silver resistant strain pMG101, and *E. coli*, silver sensitive strain R906, were obtained from Prof. S. Silver, University of Illinois at Chicago. In 16 tests, the two strains were biochemically identical. R906 was maintained on agar of composition ($gl^{-1}$): Difco casein hydrolysate peptone (tryptic) ,10; Oxoid agar bacteriological (Agar no. 1), 15; Difco Bacto yeast extract, 5; BDH NaCl, 0.06 (1 mM). The silver resistant strain pMG101 was maintained on a medium identical except for the addition of $AgNO_3$(0.068 $gl^{-1}$, 0.4 mM). Preliminary experiments had established that the presence of chloride was necessary for a difference in silver sensitivity to be observed between R906 and pMG101. When the concentration of NaCl was varied between 1 and 40 mM, an approximately equal concentration of silver as the nitrate salt was required to inhibit growth of pMG101. The NaCl concentration (1 mM) was chosen so that the concentration of $AgNO_3$ in the plates could be kept below 1 mM. At these concentrations of $AgNO_3$, there was no precipitation and the agar plates were not apparently light sensitive.

Compounds for testing were dissolved in DMA, which is miscible with water, apart from $[Ag(bipy)_2]NO_3.2H_2O$ and 2,2'-bi which were dissolved in methanol. The final solvent concentrations in the agar plates were 1-2% (v/v) for DMA, 2.5% (v/v) for methanol. Each experiment included blanks (solvent only, no compound) which showed that DMA and methanol at these concentrations did not inhibit growth of *E. coli.*

A suspension of approximately $10^6$ organisms $cm^{-3}$ (judged by eye) was made up in distilled water and 50 μl dispensed onto each plate and spread. This inoculum size achieved semi-confluent growth in the controls. All tests were done in duplicate. The plates were incubated at 37°C for 18 hours, and then examined for growth of colonies. Each experiment was repeated at least once. $AgNO_3$ at a range of concentrations was included alongside the test compounds in each experiment.

### Results

Apart from bipy, $[Ag(bipy)_2]NO_3$ and $[Ag(depe)_2]NO_3$, all the compounds turned milky on dilution in the agar solution. Table III shows the effects of the ligands at different concentrations on growth of the two strains; Table IV the effects of the complexes. The results for $AgNO_3$ are included throughout for comparison. Table V summarises the results of the $^{31}P$-{$^1H$} NMR measurements for the phosphine complexes. Addition of water alone to DMA solutions of the complexes caused precipitation from the $[Ag(PPh_3)_2Cl]$ and $[Ag(PPh_3)_3Cl\}$ solutions, but not from the other silver phosphine/DMA solutions.

### Discussion

From Table III it can be seen that the toxicities (or otherwise) of all the ligands toward the two strains are the same. This is as expected, since there is no reason to expect a silver resistant strain to show cross resistance to one of these ligands.

Comparison of the toxicities of the silver complexes of the chelating phosphines, $[Ag(dppe)_2]NO_3$, $[Ag(dppey)_2]NO_3$ and $[Ag(depe)_2]NO_3$ toward the two strains (Table IV) shows that there is very little difference for each of the three complexes. This suggests that the mechanism of toxicity in these cases is not related to the presence of silver in the complexes. $[Ag(dppe)_2]NO_3$ is toxic at 1mM. This corresponds to a dppe concentration of 2mM, which is not toxic under these conditions (Table III). Similar comparisons can be made for dppey and depe. These show that the effect is not due to the ligands alone either. Instead, the toxicities of these complexes are likely to be properties of the complex as a whole. They are hydrophobic cations which are relatively soluble and stable

Table III Effect of ligands on growth of (a) R906 (*E. coli*, silver sensitive strain) and (b) pMG101 (*E. coli*, silver resistant strain).

| conc. (mM) | $AgNO_3$ | | dppe | | dppey | | depe | | $PPh_3$ | | 2,2'-bipyridine | |
|---|---|---|---|---|---|---|---|---|---|---|---|---|
| | a | b | a | b | a | b | a | b | a | b | a | b |
| 0.00 | + | + | + | + | + | + | + | + | + | + | + | + |
| 0.05 | + | | | | | | | | + | + | | |
| 0.1 | + | | | | | | | | + | + | + | + |
| 0.2 | * | + | + | + | | | | | + | + | * | + |
| 0.4 | - | + | + | + | | | | | + | + | - | - |
| 0.6 | - | + | + | + | | | | | + | + | | - |
| 0.8 | - | * | | | | | | | + | + | | |
| 1.00 | - | - | + | + | | | + | + | + | + | | |
| 2.00 | | | + | + | | | + | + | + | + | | |
| 3.00 | | | + | + | | | | | + | + | | |
| 4.00 | | | + | + | + | + | - | - | + | + | | |

* = occasional growth
\+ = growth
\- = no growth

Table IV Effect of silver complexes on growth of (a) R906 (*E. coli*, silver sensitive strain) and (b) pMG101 (*E. coli*, silver resistant strain)

| $[Ag^{+}]$ (mM) | $AgNO_3$ | | $[Ag(dppe)_2]NO_3$ | | $[Ag(dppey)_2]NO_3$ | | $[Ag(depe)_2]NO_3$ | | $Ag(PPh_3)_2Cl$ | | $Ag(PPh_3)_3Cl$ | | $AgNO_3PPh_3$ | | $[Ag(bipy)_2]NO_3$ | |
|---|---|---|---|---|---|---|---|---|---|---|---|---|---|---|---|---|
| | a | b | a | b | a | b | a | b | a | b | a | b | a | b | a | b |
| 0.00 | + | + | + | + | + | + | | + | + | + | + | + | + | + | + | + |
| 0.025 | | | | | | | | | + | | + | | | | | |
| 0.05 | + | | + | | | | | | * | | - | | + | | + | |
| 0.10 | + | | + | | | | + | | - | + | - | | - | + | * | + |
| 0.20 | * | | + | + | + | + | + | + | - | + | | | - | + | - | - |
| 0.40 | - | + | + | + | + | + | - | + | - | + | | + | - | - | | |
| 0.60 | - | + | + | + | - | - | - | - | | + | | + | - | - | | |
| 0.80 | - | * | + | + | - | - | - | - | | + | | + | - | - | | |
| 1.00 | - | - | - | - | - | - | - | - | | + | | + | - | - | | |
| 1.20 | | | | | | | | | | + | | + | | | | |
| 1.40 | | | | | | | | | | + | | + | | | | |
| 1.60 | | | | | | | | | | + | | | | | | |

Table V Stability of silver compounds, monitored by $^{31}P\{^1H\}$ NMR spectroscopy

| Compound | NaCl | dithiothreitol | thio-β-D-Glucose, $Na^+$ salt dihydrate |
|---|---|---|---|
| $[Ag(dppe)_2]NO_3$ | + a | + | – |
| $[Ag(dppey)_2]NO_3$ | + cloudy | + | + |
| $[Ag(depe)_2]NO_3$ | + | + | + |
| $Ag(PPh_3)_2Cl$ | - precipitated | + | - |
| $Ag(PPh_3)_3Cl$ | - precipitated | + | - |
| $AgNO_3(PPh_3)$ | - precipitated | - | - |

+ = stable

- = unstable a. ref. 38

(Table V). In common with the Au(I) and Cu(I) analogues they are known to be highly toxic to B16 melanoma and P388 cell lines *in vitro* (38,91). The mechanism whereby these complexes kill cells is likely to be the same for all, irrespective of the nature of the metal or the chelating phosphine, except insofar as these affect solubility and stability of the complex. This has been discussed in detail (91).

Silver(I) complexes of the monodentate phosphine $PPh_3$ behave very differently from their chelating counterparts. They are more readily precipitated by chloride and less stable to thiols (Table V). The three tested, $[Ag(PPh_3)_2Cl]$, $[Ag(PPh_3)_3Cl]$ and $[Ag(PPh_3)NO_3]$ are all more toxic toward *E. coli* R906 than is $AgNO_3$ itself. This is not due to toxicity by $PPh_3$, which is non-toxic at these concentrations (Table III), but is presumably due to availability of $Ag^+$ from the complex. That the silver is the active component in these cases is shown by their lesser toxicity towards the silver-resistant *E. coli* pMG101 (Table IV). The two chloride complexes are even less toxic than $AgNO_3$, but $[Ag(PPh_3)NO_3]$ was consistently more toxic than $AgNO_3$. This has the exciting implication that this complex is in part circumventing the resistance system employed by *E. coli* pMG101. However, it is not clear from the chemical properties why this should be so. It was the only one of the $PPh_3$ complexes not precipitated from DMA by water, but the proportions used for the testing plates caused the agar to appear milky, and it is reported to exchange $NO_3^-$ for $Cl^-$ in solution (89).

The bipyridyl complex is in a different category from the others in that the ligand alone is as toxic as $Ag^+$ is to the sensitive *E. coli* R906, with an MIC of ~0.4mM. The toxicity of the complex (MIC ~0.2mM) could be due solely to the ligands, since a 0.2mM solution of $[Ag(bipy)_2]^+$ is 0.4mM in bipy. It is also not entirely clear whether there is a difference between the results for the two strains. It is perhaps not surprising that this complex is bacteriocidal, since such activity is already known for bipy complexes with copper (92) and other metals (10). The activity was at first ascribed to the complex as a whole, with charge, size and liptophilicity being important factors (93). However, different rates of bacterial kill by complexes with different metal ions (94) suggested kinetic lability was also important. This is probably another example of complexes damaging bacteria by more than one mechanism.

These compounds provide good examples of Sadler's classification (95) of biologically active metal complexes into 'active complexes' (silver complexes with diphosphines), 'active metal' (silver complexes with $PPh_3$) and 'active ligand' (silver with bipyridine).

The problem of dissolving silver complexes for testing purposes is rarely discussed. Modak and Fox (32) did address this. They described dissolving AgSu in 28% ammonia which was then diluted in the nutrient broth to the required concentration. In control experiments, they found such ammoniacal solutions did not significantly affect uptake of silver by *Ps. aeruginosa* and did not alter cell function (32). In our systems, the amount of ammonia required to solubilise AgSu (to achieve silver concentrations as shown in the Tables) was toxic to the *E. coli*. We also did not proceed with testing complexes which precipitated in lumps when diluted in the nutrient broth. However, most of complexes tested did precipitate (see Results) to give homogeneous, milky solutions which were not light sensitive. This raises the question of what is meant by "concentration" in such systems. Minimum inhibitory concentrations (MICs) have regularly been quoted for AgSu which are greater than its solubility of about 3mM (e.g. ref 81) and for silver as the nitrate (see Table II) which are greater than the solubility of AgCl (about 6mM at 10°C (96)). Attempts to measure dissolved silver in testing systems have found the concentrations to be very low. Belly and Kydd (51), who tabulated concentrations of 100 and 300ppm as permitting growth of resistant strains, claimed that 300ppm of $AgNO_3$ gave rise to only 3ppb of ionic silver. Ricketts *et al* (60) used a silver-specific electrode to measure $[Ag^+]$ at different combinations of added $AgNO_3$ and NaCl. In the range 0.05-0.1M NaCl, MIC values of added $AgNO_3$ of 0.1-0.2mM corresponded to 1-3 x $10^{-9}$M $Ag^+$. The MIC did not correspond with the measured concentration of silver ions, but with the total amount of silver available (60). A similar point was made by Fox and Modak (31). They measured the bacterial binding of silver from supernatant and sediments of human serum which had been reacted with seven different silver compounds. The amount of silver bound from the supernatants was only 14.5-18.5%. Much greater amounts were bound from the sediments: 40-58% from four silver sulfonamides and 74-83% from $AgNO_3$, AgCl and AgSu (31). The implication of all these observations is that silver is not taken up from solution but from undissolved silver(I) compounds. This would also seem to be implicit in Simon Silver's hypothesis regarding the effect of $Cl^-$ on silver-resistant cells (71) (see above).

The problem of understanding the biological effects of nickel(II) may provide a parallel. Soluble Ni(II) salts damaged isolated DNA, but were not carcinogenic. Insoluble, amorphous nickel sulphides were not carcinogenic either. Insoluble, crystalline, nickel sulphides *did* transform cells but did not damage isolated DNA. Finally, Costa showed that crystalline nickel sulphide (which is negatively charged) is phagocytosed, undergoes cytoplasmic dissolution and the resultant $Ni^{2+}{}_{(aq)}$ can enter the nucleus and induce DNA strand breaks (97,98). Amorphous nickel sulphides (negatively charged) are not phagocytosed, and dissolved $Ni^{2+}{}_{(aq)}$ cannot enter the cell.

It is clear that our understanding of silver toxicity to bacteria, and of bacterial resistance mechanisms, has a long way to go. We can be sure that comparative studies of resistant and sensitive strains will provide the greatest insight. In the meantime, we have identified some silver compounds which are either toxic by other means, or can circumvent the resistance mechanisms.

### Acknowledgements

We wish to thank Professor S. Silver, University of Illinois, Chicago, for provision of bacteria and of laboratory facilities at the start of this work. We thank Dr. Angela Child, UWCC, for microbiological advice and discussions, and Mrs. Sue Richards, UWCC, for typing. JLC thanks the University of Wales for a studentship.

### References

1. E.H. Blakeney (ed) (1945) "*The History of Herodotus*" translated by G. Rawlinson; Dent, London.
2. G. Sykes (1958) "*Disinfection and Sterilization*"; Spon, London.
3. R.G. Berk (1947) Abstracts of articles on oligodynamic sterilization. Project WS 768 The Engineer Board, Corps of Engineers, U.S. Army, Fort Belvoir, Va.
4. J. Raulin (1869) *Sci. Nat.*, **11**, 93. Berk (3) Abstr. 1.
5. K.W. von Nägeli (1893) *Denschr. schweiz naturforsch Ges.* **33**, 174. Berk (3) Abstr. 5.
6. I.B. Romans (1968) in "*Disinfection, Sterilization and Preservation*" edited by C.A. Lawrence and S.S. Block; Lea and Febiger, London, pp372-400 and pp469-475.
7. W.B. Hugo and A.D. Russell (1982) in "*The Principles and Practice of Disinfection, Preservation and Sterilization*", edited by A.D. Russell, W.B. Hugo and G.A.J. Ayliffe; Blackwell Scientific, p70.
8. N. Grier in "*Disinfection, Sterillization and Preservation*" ed. S.S. Clock; lea and Febiger, Philadelphia (1983) pp 375-389.
9. J.T. Trevors, *Enzyme Microb. Technol.* (1987), **9**, 331.
10. N. Farrell, "*Transition Metal Complexes as Drugs and Chemotherapeutic Agents*" Kluwer Academic Publishers (1989), pp 208-221.
11. R.M. Slawson, H. Lee and J.T. Trevors, *Biol Metals* (1990), **3**, 151.
12. A.D. Russell and W.B. Hugo, *Prog. Med. Chem.* (1994), **31**, 351.
13. G.A. Krause (1928). Pamphlet published by Bergmann, Munich. Berk (3) Abstr. 130.
14. G. Sykes (1965) "*Disinfection and Sterilization*" 2nd edition, Spon, London.
15. J. Gibbard (1933) *Can. J. Public Health*, **24**, 96. Berk (3) Abstr. 199.
16. G. Piazza (1934) *Gion. med. militare.*, **82** 323. Berk (3) Abstr. 223.
17. K. Süpfle and R. Werner (1951). *Mikrochemie ver. Mikrochim. Acta*, **36/37**, 866-881.
18. M.T. Yahya, L.K. Landeen, M.C. Messina, S.M. Kute, R. Schulz and C.P. Gerba, *Can. J. Microbiol.* (1990), **36**, 109.
19. R.L. Woodward (1963). *J. Amer. Water Works Assoc.*, **55**, 881-886.
20. W.L. Mallman (1937) *Mich. Eng. Exp. Sta. Bull.*, **12**, 5. Berk (3) Abstr. 256.
21. L. Goodman and A. Gilman (1980) "*The Pharmacological Basis of Therapeutics*" Macmillan, New York.
22. C.M.E. Rowland-Payne, C. Bladin, A.C.F. Folchester, J. Bland, R. Lapworth and D. Lane, *Lancet*, (1992), **340**, 126.
23. A.O. Goettler, C.P. Rhoads and S. Weiss, *Am. J. Path.*, (1927), **3**, 631.
24. R.S. Gibson and C.,A. Scythes, *Biol. Trace Elem., Res.*, (1984), **6**, 105.
25. C.A. Moyer, L. Brentano, D.L. Gravens, H.W. Margraf and W.W. Monafo (1965) *Arch. Surg.*, **90**, 812.
26. J.S. Cason and E.J.L. Lowbury (1968), *Lancet*, **I**, 651-654.

27. J.P. Bull (1971), *Lancet*, **II**, 1133-34.
28. E.J.L. Lowbury (1982) in "*The Principles and Practice of Disinfection, Preservation, and Sterilization*" edited by A.D. Russell, W.B. Hugo and G.A.J. Ayliffe; Blackwell Scientific.
29. C.L. Fox Jr., (1968), *Arch. Surg.* **96**, 184-188.
30. C.L. Fox Jr., B.W. Rappole and W. Stanford, *Surg. Gynecol. Stet.*, (1969), **128**, 1021.
31. C.L. Fox Jr. and S.M. Modak, *Antimicrob. Ag. Chemother.*, (1974), **5**, 582.
32. S.M. Modak and C.L. Fox Jr., *Biochem. Parmacol.*, (1973), **22**, 2391.
33. R.J. Lancashire in "*Comprehensive Coordination Chemistry*", eds. G. Wilkinson, R.D. Gillard and J.A. McCleverty, Pergamon Press (1987) Volume 5, pp777-859.
34. J. Heukeshoven and R. Dernick, *Electrophoresis*, (1985), **6**, 103.
35. A.J. Zelazowski, Z. Gasyna and M.J. Stillman, *J. Biol Chem.*, (1989), **264**, 17091.
36. M.G. Tordi, F. Naro, R. Giordano and M.C. Silvestrini, *Biol. Met.*, (1990), **3**, 73.
37. R.C. Tilton and B. Rosenberg, *Appl. Environmen. Microbiol.*, (1978), **35**, 1116.
38. S.J. Berners-Price, R.K. Johnson, A.J. Giovenella, L.F. Faucette, C.K. Mirabelli and P.J. Sadler, *J. Inorg. Biochem.* (1988), **33**, 285.
39. A.T. Tu and J.A. Reinosa, *Biochemistry*, (1966), **5**, 3375.
40. B. Singer and H. Fraenkel-Conrat, *Biochemistry*, (1962), **1**, 852.
41. R.H. Jensen and N. Davidson, *Biopolymers*, (1966), **4**, 17.
42. H.S. Rosenkranz and S. Rosenkranz, *Antimicrob. Ag. Chemother.* (1972), **2**, 373.
43. H.S. Rosenkranz and H.S. Carr, *Antimicrob. Ag. Chemother.*, (1972), **2**, 367.
44. M.S. Wysor and R.E. Zollinhofer, *Path. Microbiol.*, (1972), **38**, 296.
45. J.E. Coward, H.S. Carr and H.S. Rosenkranz, *Antimicrob. Ag. Chemother.*, (1973), **3**, 621.
46. R.M.E. Richards, *Microbios*, (1981), **31**, 83.
47. R.C. Charley and A.T. Bull, *Arch. Microbiol.*, (1979), **123**, 239.
48. T. Pümpel and R. Schinner, *Appl. Microbiol. Biotechnol.*, (1986), **24**, 244.
49. P.A. Goddard and A.T. Bull, *Appl. Microbiol. Biotechnol.*, (1989), **31**, 314.
50. F.D. Pooley, *Nature*, (1982), **296**, 642.
51. R.T. Belly and G.C. Kydd, *Dev. Ind. Microbiol.*, (1982), **23**, 567.
52. C. Haefeli, C. Franklin and K. Hardy, *J. Bacteriol.*, (1984), **158**, 389.
53. W. Ghandour, J.A. Hubbard, J. Diestung, M.N. Hughes and R.K. Poole, *Appl. Microbiol. Biotechnol.*, (1988), **28**, 559.
54. G.M. Gadd, O.S. Laurence, P.A. Briscoe and J.T. Trevors, *Biol. Met.*, (1989), **2**, 168.
55. M.E. Starodub and J.T. Trevors, *J. Med. Microbiol.*, (1989), **29**, 101.
56. R.W. Traxler and E.M. Wood, *J. Ind. Microbiol.*, (1990), **6**, 249.
57. M.E. Starodub and J.T. Trevors, *J. Inorg. Biochem.*, (1990), **39**, 317.
58. J.T. Trevors and M.E. Starodub, *Curr. Microbiol.*, (1990), **21**, 103.
59. M.E. Starodub and J.T. Trevors, *Biol. Metals*, (1990), **3**, 24.
60. C.R. Ricketts, E.J.L. Lowbury, J.,C. Lawrence, M. Hall and M.D. Wilkins, *Brit. Med. J.*, (1970), 444.
61. J. Yudkin, *Enzymologia*, (1937), **2**, 161.
62. P.J. Snodgrass, B.I. Vallee and F.L. Hoch, *J. Biochem.*, (1960), **235**, 504.
63. M.K. Rayman, T.C.Y. Lo and B.D. Sanwal, *J. Biol. Chem.*, (1972), **247**, 6332.
64. P.D. Bragg and D.J. Rainnie, *Can. J. Microbiol.*, (1974), **20**, 883.
65. J.A.M. Hubbard, M.N. Hughes and R.K. Poole, *FEBS Lett.*, (1983), **164**, 241.
66. H.S. Rosenkranz, J.E. Coward, T.J. Wlodkowski and H.S. Carr, *Antimicrob.* Ag. Chemother., (1974), **5**, 199.
67. G.L. McHugh, R.C. Moellering, C.C. Hopkins, M.N. Swartz, *Lancet*, (1975), **I**, 235.
68. D.I. Annear, B.J. Mee and M. Bailey, *J. Clin. Pathol.*, (1976), **29**, 441.
69. A.O. Summers, G.A. Jacoby, M.N. Swartz, G. McHugh and L. Sutton, in "*Microbiology-1978*" ed. D. Schlessinger, Am. Soc. for Microbiol., Washington (1978).
70. A.T. Hendry and I.O. Stewart, *Can. J. Microbiol.*, (1979), **25**, 916.
71. S. Silver, in "*Molecular Biology, Pathogenicity and Ecology of Bacterial Plasmnids*", eds. S.B. Levy, R.C. Clares and E.L. Koenig, Plenum. New York (1981), pp179-189.
72. P. Kaur and D.V. Vadehra, *Antimicrob. Ag., Chemother.*, (1986), **29**, 165.
73. R.M. Slawson, E.M. Lohmeier-Vogel, H. Lee and J.T. Trevors, *BioMetals.*, (1994), **7**, 30.
74. L.M. Deshpande and B.A. Chopade, *BioMetals.*, (1994), **7**, 49.
75. R.A. Pledger and H. Lechevalier, *Antiobio. Chemother.*, (1956), **6**, 120.
76. J.S. Cason, D.M. Jackson, E.J.L. Lowbury and C.R. Ricketts, *Br. Med. J..*, (1966), **2**, 1288-1294.
77. E.J.L. Lowbury, J.R. Babb, K. Bridges and D.M. Jackson (1976), *Brit. Med. J.*, **1**, 493-496.
78. D.I. Annear, N.J. Mee and M. Bailey, *J. Clin. Pathol.*, (1976), **29**, 441.

79. K. Bridges, A. Kidson, E.J.L. Lowbury and M.D. Wilkins, *Br. Med J.*, (1979), **1**, 446.
80. H. Nakahara, I. Yonekura, A. Sato, K. Moriyama, K. Yukata, T. Mori and M. Chino, *Water Sc. Technol.*, (1989), **21**, 275.
81. H.S. Carr, T.J. Wlodkowski and H.S. Rosenkranz, *Antimicrob. Ag. Chemother.*, (1973), **4**, 585.
82. R.M. Slawson, J.T. Trevors and H. Lee, *Arch. Microbiol.*, (1992), **158**, 398.
83. W.J.A. Schreurs and H. Rosenberg, *J. Bacteriol.*, (1982), **152**, 7.
84. S. Silver, R.D. Perry, Z. Tynecka and T. Kinscherf, in "*Drug Resistance in Bacteria*", ed. S. Mitsuhashi, Jpn. Sc. Soc., Tokyo (1982), pp 347-361.
85. D.P. Cook and M.F. Turner, *J. Chem. Soc. Perkin Trans. II*, (1975), 1021.
86. N.C. Baezinger and A.W. Struss, *Inorg. Chem.*, (1976), **15**, 1807.
87. A. Bult and C.M. Plug in "*Analytical Profiles of Drug Substances*", vol 13, ed. K. Florey, Academic Press, (1984), pp554-571.
88. S.J. Berners-Price, C. Brevard, A. Pagelot and P.J. Sadler, *Inorg. Chem.* (1985) **24**, 4278.
89. R.A. Stein and Carolyn Knobler, *Inorg. Chem.* (1977) **16**, 242.
90. D.P. Murtha and R.A. Walton, Inorg. Chem. (1973), **12**, 368.
91. S.J. Berners-Price and P.J. Sadler, *Struct. Bond*, (1988), **70**, 27.
92. H. Smit *et. al.*, *Antimicrob. Ag. Chemother.*, (1980), **18**, 249.
93.. F.P. Dwyer, I.K. Reid, A. Shulman, G.M. Laycock and S. Dixson, *Aust. J. exp. Biol. med. Scie.*, (1969), **47**, 203.
94. H.M. Butler, A. Hurse, E. Thursky and A. Shulman, *Aust. J. exp. Biol. Med. Sci.*, (1969), 47, 541.
95. P.J. Sadler, personal communication.
96. CRC Handbook of Chemistry and Physics, Student Edition (1988) CRC Press, Florida.
97. M. Costa, *Biol Trace Elem. Res.*, (1983), **5**, 285.
98. M. Costa and J.D. Heck, *Adv. Inorg. Biochem.*, (1984), **6**, 285.

